# Genetic diversity analysis of East African sorghum (Sorghum bicolor [L.] Moench) germplasm collections for agronomic and nutritional quality traits

**DOI:** 10.1016/j.heliyon.2022.e09690

**Published:** 2022-06-10

**Authors:** Charles Andiku, Hussein Shimelis, Admire I.T. Shayanowako, Prakash I. Gangashetty, Eric Manyasa

**Affiliations:** a African Center for Crop Improvement (ACCI), School of Agricultural, Earth and Environmental Sciences, University of KwaZulu-Natal, Private Bag X01, Scottsville, 3209, Pietermaritzburg, South Africa; b National Semi-Arid Resources Research Institute (NaSARRI), P.O. Box 56, Soroti, Uganda; c Busitema University, Faculty of Agriculture and Animal Sciences, Department of Crop Production and Management, P.O. Box 236, Tororo, Uganda; d International Crops Research Institute for the Semi-Arid Tropics (ICRISAT)-Niger, BP 12404, Niamey, Niger; e International Crops Research Institute for the Semi-Arid Tropics (ICRISAT)-Kenya, P.O. Box 39063, Nairobi, Kenya

**Keywords:** East Africa, Genetic diversity, Qualitative and quantitative traits, Quality breeding, *Sorghum bicolor*

## Abstract

Breeding for climate-resilient, high-yielding, and nutrient-rich sorghum cultivars is essential for sustainable food systems and enhanced livelihoods in sub-Saharan Africa. Therefore, this study aimed to determine the genetic diversity among East African sorghum germplasm collections through agronomic and nutritional quality traits to select promising lines for direct production or breeding. A collection of 348 sorghum germplasm was field evaluated at two locations in Uganda using an augmented design, and grain iron (Fe) and zinc (Zn) contents were profiled. Data were collected on 20 sorghum agro-morphological traits and Fe and Zn compositions. A significant (P ≤ 0.05) variation was detected amongst the test genotypes for all the assessed traits, suggesting the presence of sufficient genetic diversity for selection. High heritability (H^2^ > 0.60) and genetic advance as percent of the mean (GA >20%) were computed for grain yield, Zn content, and selected agronomic traits, ensuring genetic gains through selection. A significant positive correlation was recorded between Fe and Zn concentrations (r = 0.32, P < 0.001), allowing simultaneous selection for the two nutrient compositions. Cluster analysis based on phenotypic traits resolved the test sorghum genotypes into four distinct genetic groups. Six genotypes with superior agronomic traits and high Fe and Zn contents were identified for production or potential parents for quality breeding. Overall, the current study found considerable genetic variation among East African sorghum germplasm collections for strategic conservation and breeding in Uganda or similar agro-ecologies.

## Graphical abstract

**Figure:**
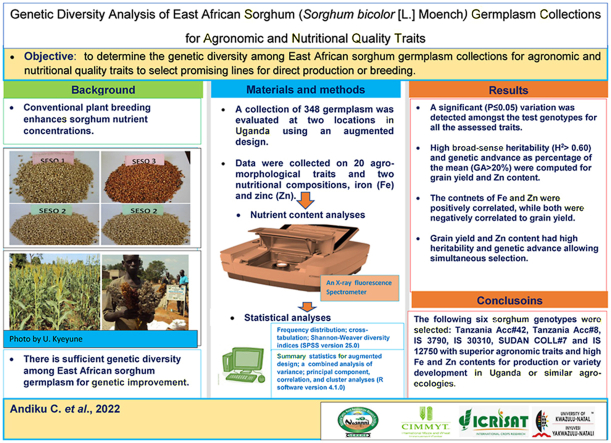

**Core ideas**

• Conventional plant breeding enhances sorghum nutrient concentrations.
• There is adequate genetic diversity among East African sorghum germplasm for breeding.
• In the assessed sorghum populations, grain Fe and Zn contents positively correlated but negatively correlated with grain yield.
• Grain yield and Zn content had high heritability and genetic advance.
• Six genotypes had high grain Fe and Zn contents among the assessed sorghum germplasm for production or genetic improvement.

## 1 Introduction

Sorghum [*Sorghum bicolor* (L.) Moench, 2n = 2x = 20] is the fifth most important cereal crop produced after rice, wheat, maize, and barley globally (FAOSTAT, 2021). Sorghum is a vital food staple and an essential source of human nutrition for millions of people in developing countries (Kumar et al., 2013b). It thrives under low soil moisture and poor soil fertility conditions, where other major cereal crops would fail. Sorghum is relatively tolerant to drought and heat stress making it a crop of choice in marginal agro-ecologies in sub-Saharan Africa (SSA).

Sorghum grain has appreciable levels of minerals, starch, gluten-free protein, and crude fiber (Kumar et al., 2015). The grain consists of about 65% carbohydrates and 15% total protein on a dry weight basis. The protein and starch in sorghum products have slow digestibility compared with other cereals. Hence sorghum products are ideal for people with gluten intolerance or diabetes.

Sorghum is an essential source of iron (Fe) and zinc (Zn) and possesses diverse mineral nutrients than rice and wheat (Chan et al., 2007). Some accessions possess higher Fe content of >60 mg kg^−1^ and Zn > 32 mg kg^−1^. Biofortified sorghum provides a substantial amount of Zn (13,000 to 18,000 mg/100g) and Fe (10,000 mg/100g) for children from 4 to 6 years old. Some 300g grain of biofortified sorghum can provide 39,000 to 54,000 mg Zn and 30,000 mg Fe to lactating women (Andiku et al., 2021). Iron supply is vital for producing new red blood cells, muscle growth, and brain development, while Zn is critical for embryo development, fetal growth, and milk expression in lactating mothers. A preliminary report by USAID (2021) indicated that a larger proportion of children below five years of age are stunted or malnourished in Uganda. Also, the report pointed out a higher prevalence of anaemia among children of 6–59 months of age and women of reproductive age due to micro-nutrient deficiency.

After maize and rice, sorghum is the third most important cereal crop in Uganda. It is cultivated across 470,083 ha accounting for 400,000 tons of grain production (FAOSTAT, 2021). Sorghum is cultivated for food, brewing, and feed in SSA and Asia (Andiku et al., 2021). It is the primary source of dietary calories, particularly for pregnant and lactating women and pre-school children in Uganda. Most farmers in Uganda grow unimproved sorghum varieties deficient in micronutrients such as Zn and Fe and with low yield potential. Furthermore, a lack of improved varieties and yield loss due to biotic and abiotic constraints are the major constraints to sorghum production in Uganda (Andiku et al., 2021). Therefore, there is a need to develop nutrient-dense, high-yielding sorghum varieties with farmer-preferred traits in the country.

Sorghum's nutritional quality has improved through conventional plant breeding, biotechnology, and crop management practices (Kumar et al., 2019). Biofortification through conventional breeding is cost-effective, and the new varieties and derived products are relatively widely adopted. The International Crops Research Institute for Semi and Arid Tropics (ICRISAT)/India has recently developed and released biofortified sorghum varieties using the conventional breeding method. The released biofortified sorghum varieties expressed higher grain Zn and Fe concentrations varying from 50 to 60% than the standard commercial cultivars (Janila et al., 2018; Kumar Ashok et al., 2018). In 2018, ICRISAT/Nigeria released the first two biofortified sorghum varieties in Africa. The released varieties had three times higher Fe concentrations and better grain yield (2.4–2.8 t ha^−1^) than the traditional sorghum cultivated in the region (Reddy and Reddy, 2019). The sorghum genetic diversity present in East Africa has not been explored to select unique genetic stocks for cultivar development with enhanced nutrition content and farmer-preferred attributes.

Phenotypic, genotypic, and biochemical markers have been used to assess the genetic diversity of sorghum. Phenotypic or agro-morphological traits are relatively simple and cost-effective to measure and valuable for ideotype breeding. However, phenotypic selection for agronomic and nutritional traits is subject to genotype, environment, and genotype × environment interaction effects. Therefore, a combination of phenotypic, genetic, and biochemical markers and multi-environmental evaluations is required for precision genotype selection and accelerated breeding. Various biochemical markers such as simple staining procedures and complex analytical methods have been used for sorghum nutrient profiling. Staining techniques provide a crude estimation of micro-nutrients, while analytical methods such as X-ray fluorescence spectrometer (XRF) are robust and preferred for micronutrient analysis (Kumar et al., 2019). The XRF technology has proven to be an accurate and more rapid method to determine the nutrient profiles of cereal grain than the wet chemistry procedures (Guild and Stangoulis, 2021). The XRF technology can simultaneously determine multiple nutrient contents at low costs in a large sample size.

Previous studies have reported the presence of significant genetic variation among sorghum genetic resources from East Africa for agronomic and quality traits (Akatwijuka et al., 2016; Kataka et al., 2018; Kiprotich et al., 2015; Olweny et al., 2015; Salih, 2011). However, these studies either used limited accessions, focused on yield-related agronomic traits, and neglected nutritional quality traits. The genetic diversity of East African sorghum collection should be assessed with nutritional quality and farmer-preferred traits using the representative number of germplasm. No study was conducted using a higher number of East African sorghum germplasm to unravel the genetic diversity while targeting Fe and Zn content and agronomic traits.

In Uganda, sorghum quality breeding has received little research and development support compared with the major staple crops. The genetic variation for quality traits in Ugandan sorghum germplasm and the existing diverse sorghum germplasm of East African collections is yet to be explored using agronomic and nutritional quality traits for breeding. Therefore, the objective of this study was to determine the genetic diversity present among East African sorghum germplasm collections for agronomic and nutritional quality traits to select promising lines for direct production or breeding. Information presented in the study serves as baseline data for nutritional quality improvement in the country or similar agro-ecologies in East Africa.

## 2 Materials and methods

### 2.1 Germplasm source and experimental design

The study used 348 sorghum genotypes collected from East Africa. Twenty-six genotypes were acquired from the National Semi-Arid Resources Research Institute (NaSARRI)/Uganda, while four check varieties released in Uganda were included. The remaining germplasm were introductions from ICRISAT/Kenya as follows: 318 landrace collections from East Africa, including from Kenya (232), Tanzania (49), and South Sudan (37). However, three genotypes (1 from South Sudan and 2 from Kenya) were excluded due to poor germination. As described below, the nutritional quality traits (i.e. Fe and Zn) were assayed from 334 sorghum genotypes. The description of the germplasm used in the study is presented in Table 1. The germplasm collections were field evaluated using a 22 × 20 augmented block design across two locations in Uganda. The experimental unit consisted of a 2.4 m width by 2 m long plot with sorghum genotypes planted using an inter-row spacing of 0.6 m and intra-row spacing of 0.2 m. The experimental units at all locations were maintained following the standard agronomic practices of sorghum in Uganda (Lubadde et al., 2019).

**Table 1:** The 334 accessions used in the study with their source and resolved genetic clusters when assessed using 11 quantitative traits across two locations in Uganda.

Accessions	Source	No.		Cluster
G1(104GRD), G13 (FRAMIDA), G22 (GBK-051462), G40 (GBK-051499), G49 (GBK-051515), G50 (GBK-051518), G52 (GBK-051520), G53 (GBK-051521), G54 (GBK-051522), G56 (GBK-051524), G57 (GBK-051525), G58 (GBK-051526), G61 (GBK-051530), G63 (GBK-051540), G64 (GBK-051542), G66 (GBK-051546), G71 (GBK-051563), G82 (GBK-051587), G83 (GBK-051589), G85 [GBK 000442A (KSG 96)], G86 (GBK 000445), G88 (GBK 000447), G89 (GBK 00046), G94 (GBK 000947), G99 (GBK 000955), G102 (GBK 000965), G113 (GBK 00367), G115 (GBK 026999), G117 (GBK 032204), G119 (GBK 032248), G121 (GBK 034596), G122 (GBK 034598), G126 (GBK 034640), G128 (GBK 034691), G131 (GBK 034699), G147 (GBK 043040), G157 (GBK 043322), G169 (GBK 043975), G171 (GBK 043987), G179 (GBK 044111), G180 (GBK 044112), G182 (GBK 044117), G193 (GE35/1/2013A), G205 (ICSB 735), G210 (IESV23007DL), G211 (IESV92021DL), G213 (IESV9204/SH), G217 (IS 26962), G219 (IS 26962-2), G220 (IS 26962-3), G238 (NTJ2), G245 (SILA), G286 (SV4), G333 (WAGITA)	ICRISAT/Kenya	54		1
G256 (SUDAN COLL # 17 AKWAR ACHOT), G275 (SUDAN COLL # 40 LOWOI KUDO PAYAM), G276 (SUDAN COLL # 41 LODUDU)	Sudan	3		
G292 (Tanzania Acc#15), G293 (Tanzania Acc#16), G294 (Tanzania Acc#17), G295 (Tanzania Acc#18), G296 (Tanzania Acc#19), G297 (Tanzania Acc#2), G299 (Tanzania Acc#21), G304 (Tanzania Acc#28), G305 (Tanzania Acc#29), G310 (Tanzania Acc#34), G312 (Tanzania Acc#36), G313 (Tanzania Acc#37), G314 (Tanzania Acc#38), G317 (Tanzania Acc#40), G318 (Tanzania Acc#41), G324 (Tanzania Acc#47), G326 (Tanzania Acc#49), G328 (Tanzania Acc#50)	Tanzania	18		
G2 (ASARECA13-1-1), G3 (ASARECA-18-3-1), G9 [NAROSORG2 (Check3)], G10 [NAROSORG3 (Check4)], G12 (Epuripur), G184 (GE/11/3/2013A/S1), G185 [GE/17/1/2013A (NAROSORG4)], G186 (GE/30/1/2013A), G190 (GE30/2/2013A), G239 (PATO), G242 (Seredo), G244 (SES0 2), G247 (SRS1108/SE3/2014A/E), G248 (SRS2708/5/2013A), G249 (SRS2708/8/2013A)	Uganda	15		
G14 (GADAMXIS8193), G15 (GAO10/010/SE1/2013A), G16 (GBK-051450), G17 (GBK-051452), G19 (GBK-051455), G23 (GBK-051466), G25 (GBK-051471), G27 (GBK-051473), G30 (GBK-051479), G31 (GBK-051481), G34 (GBK-051488), G36 (GBK-051494), G37 (GBK-051496), G38 (GBK-051497), G42 (GBK-051503), G44 (GBK-051507), G45 (GBK-051508), G48 (GBK-051513), G51 (GBK-051519), G62 (GBK-051532), G67 (GBK-051549), G73 (GBK-051565), G76 (GBK-051572), G77 (GBK-051576), G79 (GBK-051580), G80 (GBK-051581), G81 (GBK-051585), G84 (GBK-051591), G87 (GBK 000446), G96 (GBK 000950), G98 (GBK 000953), G100 (GBK 000959), G108 (GBK 000983), G111 (GBK 000998), G112 [GBK 000998 (KSG 225)], G118 (GBK 032222), G124 (GBK 034636), G125 (GBK 034639), G127 (GBK 034674), G129 (GBK 034692), G132 (GBK 034723), G134 (GBK 034758), G135 (GBK 036683), G137 (GBK 040563), G138 (GBK 040581), G139 (GBK 042990), G143 (GBK 043016), G151 (GBK 043097), G153 (GBK 0431409), G154 (GBK 043175), G156 (GBK 043321), G160 (GBK 043732), G161 (GBK 043735), G163 (GBK 043738), G164 (GBK 043911), G167 (GBK 043962), G168 (GBK 043967), G172 (GBK 043988), G174 (GBK 044048), G196 (ICSA 58), G198 (ICSA 735), G203 (ICSB 58), G207 (ICSR 14001), G209 (ICSR 93034), G212 [IESV92038/2SH (SSGE208/S1)], G218 (IS 26962-1), G221 (IS 26962-4),G222 (IS 30310), G228 (IS 5308), G236 (NAKHADADO), G334 (WAHI)	ICRISAT/Kenya	71		2
G251 [SUDAN COLL.5 (MAJOLDI)], G258 (SUDAN COLL # 2 LODOKA), G261 (SUDAN COLL # 23 AMACHIHA), G264 (SUDAN COLL # 27 IBURSAR), G270 (SUDAN COLL # 35 NDUMUTUK), G271 (SUDAN COLL # 36 LOBUHETI), G273 (SUDAN COLL # 39 LOLIKITHA), G280 [SUDAN COLL# 3 LODOKA (White)], G283 [SUDAN COLL.# 9 MERESE (Brown/Red)], G285 (SUDAN COLL.MAKWACH)	Sudan	10		
G290 (Tanzania Acc#13), G302 (Tanzania Acc#26), G306 (Tanzania Acc#30), G307 (Tanzania Acc#31), G308 (Tanzania Acc#32), G309 (Tanzania Acc#33), G311 (Tanzania Acc#35), G315 (Tanzania Acc#39), G319 (Tanzania Acc#42), G321 (Tanzania Acc#44) G323 (Tanzania Acc#46), G325 (Tanzania Acc#48)	Tanzania	12		
G4 (ASERECA15-3-1), G5 (ASERECA24-4-1), G7 [SESO1 (Check1)], G8 [SESO3 (Check2)], G187 (GE16/3/2013A/S2), G189 (GE25/1/2013A), G191 (GE30/5/2013A), G233 (LULU-D), G237 [NAROSORG1 (ICSR 160)], G241 (Sekedo), G243 (Serena)	Uganda	11		
G11 (EC 722446), G26 (GBK-051472), G28 (GBK-051475), G65 (GBK-051544), G68 (GBK-051551), G92 (GBK 000936), G93 (GBK 000946), G97 (GBK 000951), G103 (GBK 000970), G104 (GBK 000973), G107 (GBK 000979), G120 (GBK 032358), G130 (GBK 034698), G140 (GBK 042991), G141 (GBK 042992), G142 (GBK 042998),G146 (GBK 043025), (G155GBK 043312), G162 (GBK 043737), G166 (GBK 043957), G177 (GBK 044079), G178 (GBK 044083), G194 (ICSA 258), G195 (ICSA 434), G200 (ICSB 257), G201 (ICSB 258), G204 (ICSB 636), G208 (ICSR 15014),G215 (IS 2263), G216 (IS 23680), G224 (IS 33844), G225 (IS 3696), G226 (IS 3790), G229 (IS 5427), G231 (IS 6413)	ICRISAT/Kenya	35		3
G253 [SUDAN COLL # 11 DERI(Jeri)], G259 [SUDAN COLL # 20 MITEEN (Okoro)], G260 (SUDAN COLL # 21 AMACHINA), G262 (SUDAN COLL # 24 ATHATI), G263 (SUDAN COLL # 25 NATARI), G265 (SUDAN COLL # 29 BURJALURE), G266 (SUDAN COLL # 30 GWADA), G267 (SUDAN COLL # 31 LODOKA), G278 (SUDAN COLL.26 NACHOT), G281 (SUDAN COLL# 6 MADENGE), G282 (SUDAN COLL# 7 LODOKA)	Sudan	11		
G287 (Tanzania Acc#10), G289 (Tanzania Acc#12), G301 (Tanzania Acc#23), G316 (Tanzania Acc#4), G330 (Tanzania Acc#7), G331 (Tanzania Acc#8)	Tanzania	6		
G188 (GE16/4/2013A)	Uganda	1		
G6 (BRAHN), G18 (GBK-051453), G20 (GBK-051456), G21 (GBK-051461), G23 (GBK-051469), G29 (GBK-051477), G32 (GBK-051482), G33 (GBK-051484), G35 (GBK-051492), G39 (GBK-051498), G41 (GBK-051502), G43 (GBK-051506), G46 (GBK-051509), G47 (GBK-051512), G55 (GBK-051523), G59 (GBK-051527), G60 (GBK-051528), G69 (GBK-051561), G70 (GBK-051562), G72 (GBK-051564), G74 (GBK-051567), G75 (GBK-051569), G78 (GBK-051578), G90 (GBK 000929), G91 (GBK 000932), G95 (GBK 000949), G101 (GBK 000963), G105 (GBK 000975), G106 (GBK 000977), G109 (GBK 000990), G110 (GBK 000996), G114 (GBK 00441), G116 (GBK 027224), G123 (GBK 034635), G133 (GBK 034724), G136 (GBK 0405565), G144 (GBK 043018), G145 (GBK 043024), G148 (GBK 043060), G149 (GBK 043061), G150 [GBK 043062 (KSV 198)], G152 (GBK 043102), G158 (GBK 043401), G159 (GBK 043723), G165 (GBK 043924), G170 (GBK 043977), G173 (GBK 043989), G175 [GBK 044048 (KSG 229)], G176 (GBK 044078), G181 (GBK 044116), G183 (GBK 044589), G197 (ICSA 636), G199 (ICSA 749),G202 (ICSB 434), G206 (ICSB 749), G214 (IS 12750), G223 (IS 3283), G227 (IS 4688), G230 (IS 5476), G232 (KALID), G234 (M35-1), G235 (N13), G246 (SPV 1411)	ICRISAT/Kenya	63		4
G252 (SUDAN COLL # 1 LANDI-White), G254 (SUDAN COLL # 12 OLERERE), G255 (SUDAN COLL # 13 OLODIONG), G257 (SUDAN COLL # 18 DERI), G268 (SUDAN COLL # 33 NOHONYEK HOHORO), G269 (SUDAN COLL # 34 NOLOKIDOK), G272 (SUDAN COLL # 37 KODO KINE), G274 (SUDAN COLL # 4 JERI), G277 (SUDAN COLL # 8 JERI), G279 [SUDAN COLL# 10 MERESE (Light Brown)], G284 (SUDAN COLL.14)	Sudan	11		
G288 (Tanzania Acc#11), G291 (Tanzania Acc#14), G298 (Tanzania Acc#20), G300 (Tanzania Acc#22), G303 (Tanzania Acc#27), G320 (Tanzania Acc#43), G322 (Tanzania Acc#45), G327 (Tanzania Acc#5), G329 (Tanzania Acc#6), G332 (Tanzania Acc#9)	Tanzania	10		
G192 (GE30/7/2013A), G240 (PATOXWadAkraH1/1/-11), G250 (SSEA52-1)	Uganda	3		
**Other eleven accessions used during the study (not used for clustering)**				
G335 (GBK 034762), G336 (GBK 043991), G337 (GBK 044071), G338 (GBK-051500), G339 (ICSA 257), G340 (IS 40816), G341 (IS 5514)	ICRISAT/Kenya			
G342 (SUDAN COLL # 13 OLODIONG)	Sudan			
G343 (Tanzania Acc#24), G344 (Tanzania Acc#25), G345 (Tanzania Acc#3)	Tanzania			

### 2.2 Study locations

The study was conducted at two sites in Uganda, including the National Semi-Arid Resources Research Institute (1°35′N 33°35′E) and Abi Zonal Agricultural Research Development Institute (AbiZARDI) (3°4.58′N 30°56′E) during the summer growing seasons of 2019 and 2020. The study locations represent the major sorghum production agro-ecologies in Uganda. NaSARRI is located in the east of Uganda at an altitude of 1140 m above sea level (m.a.s.l) and receives total annual rainfall ranging between 900 to 1000 mm with a bimodal distribution and has a mean annual temperature of 26 °C. AbiZARDI lies in the northwest of Uganda at an altitude of 1,215 m.a.s.l, receives an average rainfall of 1,404 mm per year, has a unimodal rainfall pattern, and a mean annual temperature of 23.9 °C. Both sites are characterized by sandy loam soils (Salvaradjou et al., 2005).

### 2.3 Data collection

Data were collected on 20 agro-morphological traits, including seven qualitative and 13 quantitative traits following the descriptors for sorghum (IBPGR, 1993), which are summarised in Table 2. Five selected and tagged panicles per genotype were covered with a brown bag before flowering to minimize cross-pollination and harvest true-to-type seeds for grain Fe and Zn analysis. Harvesting was done manually at physiological maturity at all the study sites.

**Table 2:** Traits assessed during the study with corresponding measurements and units.

Trait	Description	Unit
Quantitative traits		
Days to 50% flowering	Days from sowing to 50% plant flowering	Days
Days to 75% maturity	Days from sowing to 75% physiological maturity based on a dark layer at the tip of the sorghum kernel	Days
Grain filling duration	Difference between the number of days to maturity and days to 50% flowering	Days
Plant height	Measured from ground level to the tip of the panicle at physiological maturity	Cm
Rachis number	Number of rachis per panicle	Number
Panicle length	From the lower panicle branch to the tip of the panicle at maturity	Cm
Panicle width	Width of the panicle in a natural position at the widest part	Cm
Dry panicle weight	Weight of dry panicle before threshing	G
100 seed weight	Weight of 100 seeds at 12.5% moisture content	G
Grain yield	Grain weight per plot at 12.5% moisture content	G
Qualitative traits		
Agronomic score	Visual rating at the vegetative stage; 1 to 3 scales, where 1 indicates Poor, 2 = Average, and 3 = Good	Codes
Panicle exsertion	Length of peduncle from flag leaf to the base of inflorescence; 1=<2 cm, slightly exserted; 2 = 2–10 cm, exserted; 3=>10 cm, well exserted; 4 = Peduncle recurved	Codes
Grain color	1 = White, 2 = Yellow 3 = Red, 4 = Brown, 5 = Buff, 6 = Others (specify)	Codes
Glume color	1 = Sienna (yellow group), 2 = Mahogany (greyed-orange group), 3 = Red, 4 = Black, 5 = Purple, 6 = White, 7 = Grey 8 = Others (specify)	Codes
Leaf midrib color	1 = Pale green, 2 = White 3 = Green, 4 = Purple, and 5 = Colorless	Codes
Inflorescence compactness	1 = Compact, 2 = Semi-compact, 3 = Loose, and 4 = Semi-loose	Codes
Inflorescence shape	1 = Erect, 2 = Drooping, 3 = Elliptic, and 4 = Oval	Codes
Glume covering	Amount of grain covered by glum; 1 = 25%, 2 = 50%, 3 = 75%, 4 = 100% or grain fully covered, and 5 = Glumes longer than the grain	Codes
Awns	1 = present or 2 = absent at maturity	Codes
Stay green	Visual rating at physiological maturity; 1 to 5 scales, where 1 = Very slightly senescent; 2 = Slightly senescent; 3 = Intermediate (about half of the leaves dead); 4 = Mostly senescent; and 5 = completely senescent (plant leaves and stalk dead)	Codes

After harvesting, panicles were sun-dried to obtain a seed moisture content of 12.5%. Panicles from each plot were later threshed, winnowed, and seeds packed. A composite seed sample of 100g from each accession was weighed, tagged, and packed in clean cloth bags and sent to ICRISAT/Niamey in Niger for grain micronutrient analysis and to profile the grain Fe and Zn content by an X-ray fluorescence spectrometer (XRF) method.

### 2.4 Data analysis

Data collected on qualitative traits were subjected to analyses, including frequency distribution, cross-tabulation, and Shannon-Weaver diversity indices. The statistical package for social scientists (SPSS) version 25.0 (George and Mallery, 2019) and Microsoft Excel were used to capture and analyze data. The qualitative phenotypic diversity among sorghum accessions was estimated using Shannon-Weaver diversity indices (*H′)* based on the frequency data as described by Jain et al. (1975). The diversity index *H′* and Shannon's equitability, *E*_*H*_,was calculated as:

*H = ΣP*_*i*_*log*_*e*_*P*_*i*_, where*: H =* Shannon diversity index*, P*_*i*_ = proportion of accessions in the *i*^th^ class of an n class trait in a population.

*E*_*H*_$=\frac{H\;}{H\text{max}}$, where*: H*_max_ = ln*S*, *S =* total number of species in the community (richness).

Data collected on quantitative traits were subjected to statistical analyses using the descriptive statistics for each experimental site separately using the R package for augmented design (Aravind et al., 2021). Shoot fly count and stem borer count data were normalized by square root transformation before analysis of variance. A combined analysis of variance was conducted using pooled data of adjusted mean values across the experimental sites using the R software version 4.1.0 (R Core, 2021). The phenotypic, genotypic, and environmental variances denoted as σ^2^p, σ^2^g, and σ^2^e, respectively, were computed from the expected mean square values as described by Federer and Searle (1976). The phenotypic, genotypic, and environmental coefficients of variation denoted as PCV, GCV, and ECV, respectively, were calculated according to Muchira et al. (2021). The GCV and PCV estimates were classified according to Sivasubramanian and Madhavamenon (1973) scales, where values of 0–10% represented low, 11–20% moderate, and >20% high. Broad-sense heritability (H^2^) values were estimated according to Lush (1940), and values were classified according to Robinson (1966), where H^2^ values of 0–30% represented low, 31–60% medium, and >61% high. The expected (predicted) genetic advance was calculated as the product of broad-sense heritability and phenotypic standard deviation at a selection intensity of 5% according to Johnson et al. (1955) as follows:

GA = H^2^ *x σ*_*p x*_ *k,* where: GA = expected genetic advance; k = selection differential in standardized units or a selection intensity of 5% = 2.056; and σ_p_ = phenotypic standard deviation. The genetic advance was expressed as a percent of the mean of the unselected parental population, GA% = $\frac{\text{GA}}{\bar{x}}\text{x}\;100$, where: GA% = Genetic advance as percent of the mean, $\bar{x}$ = Population mean for the trait considered. The GA% values were classified following Johnson et al. (1955) as follows: values of 0–10% denoted low, 11–20% moderate, and >20% high.

Based on the pooled data, principal component, correlation, and cluster analyses were conducted with the R software version 4.1.0. Principal component analysis was computed to determine the major components that could group the agro-morphological traits and examine each trait's percentage contribution to total genetic variation. Correlation coefficients among the studied traits were performed using the matrix procedure CORR and the optional PEARSON method in R software. Phenotypic cluster analysis was done using the unweighted pair group method with arithmetic mean (UPGMA) based on Euclidean distance matrix (Mayor et al., 2004; Spark, 1973) to obtain a K-means cluster.

## 3 Results

### 3.1 Variation for qualitative traits

The test genotypes showed marked genetic variations for qualitative traits across the study sites (Table 3). Significant genotype variation (p < 0.001) was recorded for all the assessed qualitative traits such as glume color, leaf midrib color, grain color, inflorescence shape and compactness, glume covering, and presence of awn on the panicle (Table 3). The white leaf midrib-color (displayed by 70.4% of the assessed genotypes) and naked or awnless panicle at maturity (92.5%) were the most predominant traits in the assessed sorghum accessions. Sixty percent of the genotypes had 25% of their grain covered by glumes, followed by a glume coverage of 50% (32.2%) and 75% (5.5%). The predominant inflorescence shape was of drooping type (44.9%), followed by the elliptic (42.0%), oval (11.3%), and erect (1.7%) types. About 48.4% of accessions had a loose inflorescence, followed by a semi-compact (30.7%) type. Only 20.6% of the accessions had compact inflorescence, while 0.3% had semi-loose inflorescence. The collections comprised red glume (75.7%) and yellow glume (18.2%) types. Diverse grain colors were observed in the germplasm, with the most prevalent brown seed color at 35.4%. Other grain colors present in the germplasm were red (27.3%), yellow (18.8%), white (16.5%), and buff (a light brown to the yellow group) (2.0%). Most sorghum genotypes (50.5%) had exerted inflorescence (2–10 cm between ligule and inflorescence base) followed by well-exserted inflorescence; >10 cm between ligule and inflorescence base (35.9%), peduncle recurved inflorescence exsertion, i.e., inflorescence below ligule and clearly exposed splitting the leaf sheath (8.2%), and slightly exserted inflorescence exsertion, i.e., <2 cm but ligule of flag leaf below inflorescence base panicle exsertion length (5.5%). A large collection of the genotypes (62.4%) had a good agronomic score, while 31.4% of the genotypes had an average agronomic score (Table 3). Most of the collections (38.8%) had intermediate senescence about half of their leaves dead, followed by mostly senescent plants (31.4%), and only 20.2% of sorghum genotypes had very slight (0.5%) to slightly (19.7%) plant leaf death and remained green. The Shannon-Weaver diversity indices (H′) resolved adequate phenotypic polymorphism in the qualitative traits with a mean value of 0.92.

**Table 3:** Shannon diversity indices, corresponding proportion (%), and significance tests for qualitative traits among 345 sorghum genotypes evaluated in two locations in Uganda.

Traits	Category	Proportion (%)	Diversity index (H′)	Shannon's equitability (*E*_*H*_)	df	Chi-square
Glume color	1	18.2	0.72	0.12	1032	2574.0∗∗∗
	2	1.2				
	3	75.7				
	4	5.0				
Leaf midrib color	1	29.6	0.61	0.1	344	858.0∗∗∗
	2	70.4				
Grain color	1	16.5	1.41	0.24	1376	3432.0∗∗∗
	2	18.8				
	3	27.3				
	4	35.4				
	5	2.0				
Inflorescence compactness	1	20.6	1.06	0.18	1032	2574.0∗∗∗
	2	30.7				
	3	48.4				
	4	0.3				
Inflorescence shape	1	1.7	1.04	0.18	1032	2574.0∗∗∗
	2	44.9				
	3	42.0				
	4	11.3				
Glume covering	1	60	0.93	0.16	1376	3432.0∗∗∗
	2	32.2				
	3	5.5				
	4	1.4				
	5	0.9				
Awn	1	7.5	0.27	0.05	344	858.0∗∗∗
	2	92.5				
Agronomic score	1	6.3	0.83	0.14	688	758.8∗
	2	31.4				
	3	62.4				
Panicle exsertion	1	5.5	1.08	0.18	1032	2203.4∗∗∗
	2	50.5				
	3	35.9				
	4	8.2				
Stay green	1	0.5	1.3	0.22	1376	3421.4∗∗∗
	2	19.7				
	3	38.8				
	4	31.4				
	5	9.7				

^a^Grain color [1 = White, 2 = Yellow 3 = Red, 4 = Brown, 5 = Buff (a light brown to yellow group), 6 = Others]; Glume color (1 = Sienna [yellow group], 2 = Mahogany (grey to orange group), 3 = Red, 4 = Black, 5 = Purple, 6 = White, 7 = Grey, 8 = Other); Leaf midrib color (1 = Pale green, 2 = White, 3 = Green, 4 = Purple, and 5 = Colorless); Inflorescence compactness (1 = Compact, 2 = Semi-compact, 3 = Loose, and 4 = Semi-loose); Inflorescence shape (1 = Erect, 2 = Drooping, 3 = Elliptic, and 4 = Oval); Glum covering (Amount of grain covered by glum; 1 = 25%, 2 = 50%, 3 = 75%, 4 = 100% or grain fully covered, and 5 = Glumes longer than grain); Awn (1 = present or 2 = absent at maturity); Agronomic score (1 = Poor, 2 = Average, and 3 = Good); Panicle exsertion (1=<2 cm, slightly exserted; 2 = 2–10 cm, exserted; 3=>10 cm, well exserted; 4 = Peduncle recurved); Stay green (Visual rating at physiological maturity; 1 to 5 scales, where 1 = Very slightly senescent; 2 = Slightly senescent; 3 = Intermediate (about half of leaves dead); 4 = Mostly senescent; and 5 = completely senescent (plant leaves and stalk dead); ∗ = P ≤ 0.05; ∗∗∗ = P ≤ 0.001; df = Degree of freedom.

Grain color recorded the highest overall diversity (H' = 1.41) in the collection followed by stay green (H' = 1.3), panicle exsertion (H' = 1.08), inflorescence compactness (H' = 1.06), inflorescence shape (H' = 1.04), glume covering (H' = 0.92), agronomic score (H' = 0.83), and glume color (H' = 0.72). The least heterogeneity was recorded for the presence of awn (H' = 0.27). The same trend was noted for Shannon's equitability, where more homogeneity was observed for grain color (E_H_ = 0.24) followed by stay green (E_H_ = 0.22) and the least recorded for awn (E_H_ = 0.05).

The analysis indicated that there were significant differences (p < 0.05) among all the genotypes, including the test populations, and check varieties for all the assessed agronomic traits except for 100 seed weight at both locations (Table 4). Test genotypes showed variation for stem borer count and 100 seed weight at the NaSARRI and AbiZARDI sites, respectively. A significant difference (P ≤ 0.05) was also recorded for the test vs. check interaction for most of the traits except for shoot fly count, stem borer count, rachis number, grain filling duration, dry panicle weight, Fe and Zn content (Table 4). A combined analysis of variance was conducted using pooled data across the experimental locations. The genotype × environment interaction effects were significant (p < 0.05) for plant height, panicle length, panicle width, rachis number, days to 50% flowering, grain filling duration, days to 75% maturity, and Zn content (Table 4). The genotype main effect was significant (p < 0.01) for all assessed traits except stem borer count, while the location effect was non-significant (p < 0.05) for plant height and rachis number.

**Table 4:** Analysis of variance for 14 quantitative traits in sorghum germplasm assessed across two locations in Uganda.

Variable	Locations and test genotypes												
	AbiZARDI					NaSARRI					Across locations		
	Genotypes	Checks	Test genotypes x Checks	Test genotypes	Block	Genotypes	Checks	Test genotypes x Checks	Test genotypes	Block	Genotype	Environment	Genotype x Environment
Df	344.00	3.00	1.00	340.00	21.00	344.00	3.00	1.00	340.00	21.00	340.00	1.00	340.00
Traits													
SF	1.47 ∗∗	10.04 ∗∗	0.02 ns	1.40 ∗∗	1.98 ∗∗	2.21 ∗∗	3.51 ∗∗	0.67 ns	2.20 ∗∗	2.37 ∗∗	1.39∗∗	42.87∗∗∗	0.82ns
SB	2.9 0∗∗	10.27 ∗∗	0.20 ns	2.84 ∗∗	9.19 ∗∗	4.54 ns	46.49 ∗∗	13.44 ns	4.14 ns	5.98 ns	2.76ns	291.09∗∗∗	1.33ns
PHT	6342.05 ∗∗	22626.75 ∗∗	395757.96 ∗∗	5053.02 ∗∗	164.64 ∗	12222.12 ∗∗	25840.11 ∗∗	860222.17 ∗∗	9607.84 ∗∗	291.01 ns	9043.30∗∗∗	719.40ns	585.50∗∗∗
PNL	49.28 ∗∗	135.51 ∗∗	64.33 ∗∗	48.48 ∗∗	4.87 ∗∗	98.87 ∗∗	90.17 ∗∗	51.85 ∗	99.09 ∗∗	13.49 ns	89.35∗∗∗	501.99∗∗∗	6.67∗
PNW	1.86 ∗∗	2.73 ∗∗	4.60 ∗∗	1.85 ∗∗	1.19 ∗	3.05 ∗∗	7.65 ∗	13.65 ∗	2.98 ∗	1.12 ns	2.24∗∗∗	3.68ns	1.12∗
RNM	330.75 ∗∗	2988.21 ∗∗	683.40 ∗	306.26 ∗∗	90.17 ns	373.60 ∗∗	2590.23 ∗∗	0.04 ns	355.14 ∗∗	51.67 ns	405.32∗∗∗	8212.24∗∗∗	101.63∗∗∗
DTF	273.54 ∗∗	699.44 ∗∗	9104.45 ∗∗	243.81 ∗∗	38.55 ∗∗	395.44 ∗∗	297.07 ∗∗	19396.02 ∗∗	340.43 ∗∗	11.66 ns	391.19∗∗∗	1058.67∗∗∗	22.68∗∗∗
GRF	41.7 0∗∗	336.74 ∗∗	0.21 ns	39.22 ∗∗	24.88 ns	49.25 ∗∗	185.92 ∗∗	316.31 ∗∗	47.26 ∗∗	8.59 ns	42.57∗∗∗	3436.06∗∗∗	18.80∗∗∗
DTM	273.64 ∗∗	72.22 ∗∗	9035.55 ∗∗	249.65 ∗∗	9.52 ns	379.96 ∗∗	156.88 ∗∗	14758.49 ∗∗	339.64 ∗∗	2.52 ns	395.75∗∗∗	681.72∗∗∗	25.75∗∗∗
DPW	2393147.66 ∗∗	12028544 ∗∗	16868375.88 ∗∗	2265614.5 ∗∗	2074289.89 ∗∗	3928965.02 ∗∗	8506370.98 ∗∗	803452.55 ns	3897768.83 ∗∗	2251930.16 ns	3246900.00∗∗∗	12105000.00∗∗∗	1014700.00ns
YLD	1192294.81 ∗∗	1841292.12 ∗∗	31030340.45 ∗∗	1098527.90 ∗∗	929840.47 ∗∗	2103236.03 ∗∗	2300756.65 ∗∗	36462105.28 ∗∗	2000437.70 ∗∗	1324233.80 ∗∗	1866943.00∗∗∗	7259871.00∗∗∗	299051.00ns
HSW	0.52 ns	1.13 ∗	4.01 ∗∗	0.51 ns	0.30 ns	0.93 ∗∗	2.82 ∗∗	7.95 ∗∗	0.89 ∗∗	0.37 ns	0.74∗∗∗	33.56∗∗∗	0.24ns
Df	333.00	3.00	1.00	329.00	21.00	333.00	3.00	1.00	329.00	21.00	329.00	1.00	329.00
Fe	91.74 ∗∗	228.53 ∗∗	710.64 ∗∗	88.61 ∗∗	56.71 ns	120.68 ∗	673.15 ∗∗	10.64 ns	115.97 ∗	100.47 ns	145.26∗∗∗	1368.92∗∗∗	77.23ns
Zn	21.44 ∗∗	62.66 ∗∗	38.22 ns	21.01 ∗∗	19.93 ∗	33.74 ∗∗	54.84 ∗	32.30 ns	33.56 ∗∗	19.76 ns	37.49∗∗∗	1055.09∗∗∗	22.85∗∗∗

^a^Df = Degree of freedom; AbiZARD = Abi Zonal Agricultural Research Development Institute; NaSARRI = National Semi-Arid Resources Research Institute; PHT = Plant height (cm); PNL = Panicle length (cm); PNW = Panicle width (cm); RNM = Rachis number; DTF = Days to 50% flowering (days); GRF = Grain filling duration (days); DTM = Days to 75% maturity (days); DPW = Dry panicle weight (kg/ha); YLD = Grain Yield (kg/ha); HSW = 100 seed weight (g); Fe = Iron (ppm); Zn = Zinc (ppm); ns = P > 0.05; ∗ = P ≤ 0.05; ∗∗ = P ≤ 0.01; ∗∗∗ = P ≤ 0.001.

### 3.2 Performance of assessed sorghum genotypes for grain yield and quality traits across locations

Based on grain yield performance, 32 genotypes had ≥12% yield gain over the best commercial check cultivar (NAROSORG3), while 80 genotypes had grain yields higher than the best commercial check cultivar (NAROSORG3) (Table 5). A mean yield of 2909.3 kg ha^−1^ was attained across the study locations. Genotype GBK 000955 had the highest grain yield of 4899.9 kg ha^−1^, which was 31.9% higher than the best commercial check cultivar (NAROSORG3). The next best-ranked genotype was GE/30/1/2013A with grain yield gain of 31.2% followed by genotypes SILA (29.6% yield gain), GBK 034699 (28.6%), GBK 044111 (28.2%), GBK 043040 (27.2%), GBK-051589 (26.3%), GBK 000445 (25.9%), GBK-051521 (25.1%), and Epuripur (24.6%). Most of the high yielding genotypes were early to medium maturing with comparatively short to medium plant height compared with the commercial check cultivars such as SESO1 and SESO3. All the 32 best performing genotypes, including the checks, had grain Fe and Zn content below the standard acceptable levels of Fe (>60 ppm) and Zn (>32ppm). Five genotypes recorded higher grain Fe concentrations. These were: Tanzania Acc#42 (with a mean Fe and Zn content of 65.5 ppm and 10.2 ppm, respectively), Tanzania Acc#8 (64.7 ppm Fe and 26.3 ppm Zn), IS 3790 (63.5 ppm Fe and 24.6 ppm Zn), IS 30310 (63.3 ppm Fe and 26.4 ppm Zn), and SUDAN COLL# 7 (61.3 ppm Fe and 25.0 ppm Zn). Genotype IS 12750 recorded a higher grain Zn concentration with a mean Zn content of 32.5 ppm (Table 6). The standard acceptable level of Fe is > 60 ppm and Zn > 32ppm for crop biofortification programs (Chapke and Tonapi, 2016). The six genotypes had relatively low grain yield levels compared to the commercial check cultivar except Tanzania Acc#42 (3315.3 kg ha^−1^) and IS 30310 (3534.2 kg ha^−1^), which had medium plant height and early to medium maturity (Table 6).

**Table 5:** Mean performance of the top 32 sorghum accessions based on higher grain yield (kg ha^−1^) and iron and zinc contents, and other quantitative agronomic attributes when evaluated across two locations in Uganda.

Genotypes	YLD	Rank	% above best check	SF	SB	PHT	PNL	PNW	RNM	DTF	GRF	DTM	DPW	HSW	Fe	Zn
Top 32 genotypes with yield gain above 12% over the best commercial check cultivar																
GBK 000955	4899.9	1	31.9	0.2	34.7	228.2	25.5	5.9	36.1	60.8	47.5	108.3	8320.0	3.0	40.5	22.2
GE/30/1/2013A	4873.0	2	31.2	5.9	17.4	175.2	21.0	6.1	27.0	68.8	38.5	107.3	6500.1	2.2	24.5	14.1
SILA	4811.7	3	29.6	8.5	15.2	144.1	25.6	5.1	58.9	74.6	34.4	109.0	9077.3	2.0	39.6	20.6
GBK 034699	4776.3	4	28.6	7.3	18.6	297.9	28.2	5.2	36.4	79.9	35.8	115.7	6370.5	2.9	42.6	17.0
GBK 044111	4763.0	5	28.2	2.8	32.5	183.3	26.1	6.0	57.1	65.1	42.1	107.3	5857.4	2.9	28.8	14.9
GBK 043040	4723.1	6	27.2	4.8	16.9	278.1	22.2	6.0	44.1	74.9	38.1	113.0	6897.0	2.9	29.2	17.0
GBK-051589	4692.2	7	26.3	3.9	30.2	184.7	20.8	7.4	44.8	72.7	38.3	111.0	9173.4	2.9	42.4	13.8
GBK 000445	4677.9	8	25.9	1.0	14.5	310.6	18.6	6.9	50.5	81.7	32.3	114.0	7477.4	2.8	33.9	10.3
GBK-051521	4646.1	9	25.1	10.0	31.6	276.2	17.9	6.9	42.4	81.4	33.6	115.0	7025.2	2.9	42.3	16.6
Epuripur	4626.9	10	24.6	5.1	5.2	183.1	21.8	5.4	39.4	75.1	35.9	111.0	7295.7	2.5	31.0	18.9
IS 26962-2	4580.8	11	23.3	1.2	8.9	185.0	27.0	6.2	45.5	73.1	38.4	111.5	6778.7	2.9	36.9	21.1
SUDAN COLL # 40 LOWOI KUDO PAYAM	4523.3	12	21.8	1.6	20.0	240.2	20.3	6.2	66.3	72.7	38.0	110.7	6086.4	2.7	34.4	17.6
GBK 044117	4498.9	13	21.1	6.0	28.2	261.5	25.1	6.6	68.3	94.4	35.0	129.5	7128.1	2.9	31.1	9.5
Tanzania Acc#38	4494.7	14	21.0	4.7	27.1	250.9	17.7	7.9	48.3	76.3	38.1	114.5	6392.1	1.8	38.3	17.4
Tanzania Acc#21	4485.6	15	20.8	2.1	52.2	258.8	18.5	9.5	47.1	61.3	49.9	111.2	6328.8	1.6	43.3	21.0
SRS2708/8/2013A	4438.6	16	19.5	5.5	21.6	181.0	24.7	6.2	48.3	69.2	39.8	109.0	7016.7	2.7	31.5	18.8
GBK 00046	4404.2	17	18.6	7.2	27.3	282.7	28.5	6.0	56.4	81.1	34.1	115.2	7600.7	1.9	49.7	17.4
SRS2708/5/2013A	4396.7	18	18.4	4.9	20.1	198.2	22.6	7.6	51.6	69.7	37.0	106.8	6562.9	2.7	34.0	15.2
GBK 034640	4393.5	19	18.3	6.9	13.4	258.8	20.5	6.2	45.0	75.2	39.0	114.2	6166.7	2.9	41.0	15.1
GBK 034598	4375.0	20	17.8	3.4	18.0	260.5	21.2	6.4	26.2	72.9	38.8	111.7	6138.1	2.7	40.7	18.6
Tanzania Acc#18	4352.9	21	17.2	4.3	25.9	257.5	23.3	5.9	57.8	76.2	38.8	115.0	6988.5	2.9	42.5	18.9
GBK-051524	4339.1	22	16.8	3.5	14.6	246.6	20.3	6.6	42.8	82.6	35.4	118.0	6623.9	2.1	33.9	17.8
IS 26962	4306.6	23	16.0	5.7	15.4	164.6	28.4	5.8	49.8	74.9	36.1	111.0	7491.6	4.4	43.5	17.7
IESV23007DL	4259.0	24	14.7	5.1	36.1	185.1	29.0	6.5	47.0	66.3	44.5	110.8	7475.1	3.0	25.5	15.6
GBK 043975	4250.1	25	14.4	1.9	38.1	260.9	18.9	6.8	46.8	77.9	37.3	115.2	6596.8	2.5	30.3	15.7
SRS1108/SE3/2014A/E	4243.3	26	14.2	1.5	27.1	199.7	22.2	6.0	43.1	65.4	41.8	107.2	7154.9	2.5	25.7	14.9
GBK-051515	4226.6	27	13.8	4.7	49.1	274.9	22.1	6.3	38.9	68.7	42.0	110.7	5977.2	2.5	44.0	20.7
Tanzania Acc#49	4217.0	28	13.5	1.8	51.1	270.5	19.7	8.7	43.0	76.6	38.1	114.8	6949.9	2.3	31.4	18.7
SES0 2	4186.3	29	12.7	2.2	20.0	152.3	23.8	4.9	36.2	77.5	35.3	112.8	7566.4	2.3	24.4	15.6
GBK 034691	4183.5	30	12.6	8.3	15.5	289.9	24.9	6.7	29.3	71.4	42.4	113.8	6997.3	2.3	39.3	15.5
GE35/1/2013A	4164.0	31	12.1	7.8	18.4	178.7	21.9	5.2	40.1	69.3	39.0	108.3	5498.6	2.2	19.9	12.1
GBK-051500	4160.8	32	12.0	7.7	29.9	287.3	18.3	7.5	68.3	82.4	36.4	118.8	5685.4	1.7		
Commercial check cultivars																
NAROSORG3	3714.1	81	0.0	4.8	31.7	212.8	23.0	6.0	61.0	71.5	37.7	109.2	6505.3	2.7	33.1	16.0
NAROSORG2	3707.1	84	-0.2	6.3	26.7	192.5	22.8	5.7	42.3	73.0	38.6	111.7	5434.0	2.3	34.5	16.6
SESO1	3298.7	132	-11.2	2.6	15.0	147.9	26.7	5.2	48.8	64.1	44.1	108.1	5550.2	2.3	33.8	18.1
SESO3	3238.1	140	-12.8	4.6	27.6	169.2	25.6	5.3	62.2	67.2	41.4	108.5	5150.5	2.1	41.4	18.3
Mean (N = 345 & 334 for Fe & Zn)	2909.3			4.4	23.6	256.6	23.7	5.8	52.0	80.3	39.7	120.1	5379.2	2.1	37.5	18.0
LSD (5%)	2687.59∗∗∗			5.36∗∗	10.67ns	187.05∗∗∗	18.59∗∗∗	2.94∗∗∗	39.60∗∗∗	38.90∗∗∗	12.83∗∗∗	39.13∗∗∗	3544.31∗∗∗	1.69∗∗∗	23.71∗∗∗	12.04∗∗∗
SE±	39.99			0.04	0.06	2.65	0.27	0.05	0.62	0.55	0.23	0.56	56.05	0.03	0.41	0.22
Std	1044.25			1.08	1.57	69.29	6.97	1.30	16.27	14.42	5.97	14.53	1463.66	0.73	10.63	5.63
CV %	35.98			51.27	32.29	26.91	29.38	22.24	31.32	17.92	15.03	12.09	27.23	34.66	28.30	31.27

^a^PHT = Plant height (cm); PNL = Panicle length (cm); PNW = Panicle width (cm); RNM = Rachis number; DTF = Days to 50% flowering (days); GRF = Grain filling duration (days); DTM = Days to 75% maturity (days); YLD = Grain Yield (kg/ha); HSW = 100 seed weight (g); Fe = Iron (ppm); Zn = Zinc (ppm); LSD = Least significant difference; SE± = Standard error of mean; Std = Standard deviation; CV = Coefficient of variation; ns = P > 0.05; ∗∗ = P ≤ 0.01; ∗∗∗ = P ≤ 0.001.

**Table 6:** The selected sorghum accessions for the best grain iron (>60 ppm) and zinc (>32ppm) concentrations and yield and related traits.

Genotypes	Fe	Zn	YLD	DPW	HSW	SF	SB	PHT	PNL	PNW	RNM	DTF	GRF	DTM
Top seven nutrient-dense selected genotypes														
Tanzania Acc#42	65.5	10.2	3315.3	6271.5	2.2	4.5	47.2	253.7	19.9	7.8	42.1	72.2	41.3	113.5
Tanzania Acc#8	64.7	26.3	579.8	1197.6	1.5	13.4	50.3	340.3	27.4	5.1	80.9	106.7	46.0	152.7
IS 3790	63.5	24.6	1332.0	4350.5	1.5	2.8	35.9	202.2	17.4	4.5	48.1	66.4	43.1	109.5
IS 30310	63.3	26.4	3534.2	6518.1	2.8	9.8	36.6	246.9	23.2	7.4	86.1	72.3	36.4	108.7
SUDAN COLL# 7 LODOKA	61.3	25.0	685.9	3110.9	2.3	11.7	27.2	422.9	35.7	5.7	41.7	95.2	41.8	137.0
IS 12750	48.2	32.5	2178.8	4424.9	2.2	3.1	24.2	270.9	23.0	5.2	57.7	75.9	37.6	113.5
ICSA 735	43.5	31.0	2741.1	6771.6	2.5	6.3	8.9	162.7	27.2	5.6	49.4	63.2	49.0	112.2
Commercial check cultivars														
SESO3	41.4	18.3	3238.1	5150.5	2.1	4.6	27.6	169.2	25.6	5.3	62.2	67.2	41.4	108.5
NAROSORG2	34.5	16.6	3707.1	5434.0	2.3	6.3	26.7	192.5	22.8	5.7	42.3	73.0	38.6	111.7
SESO1	33.8	18.1	3298.7	5550.2	2.3	2.6	15.0	147.9	26.7	5.2	48.8	64.1	44.1	108.1
NAROSORG3	33.1	16.0	3714.1	6505.3	2.7	4.8	31.7	212.8	23.0	6.0	61.0	71.5	37.7	109.2
Statistics														
Mean (N = 345 & 334 for Fe & Zn)	37.5	18.0	2909.3	5379.2	2.1	4.4	23.6	256.6	23.7	5.8	52.0	80.3	39.7	120.1
LSD 5%	23.71∗∗∗	12.04∗∗∗	2687.59∗∗∗	3544.31∗∗∗	1.69∗∗∗	5.36∗∗	10.67ns	187.05∗∗∗	18.59∗∗∗	2.94∗∗∗	39.60∗∗∗	38.90∗∗∗	12.83∗∗∗	39.13∗∗∗
SE±	0.41	0.22	39.99	56.05	0.03	0.04	0.06	2.65	0.27	0.05	0.62	0.55	0.23	0.56
Std	10.63	5.63	1044.25	1463.66	0.73	1.08	1.57	69.29	6.97	1.30	16.27	14.42	5.97	14.53
CV	28.30	31.27	35.98	27.23	34.66	51.27	32.29	26.91	29.38	22.24	31.32	17.92	15.03	12.09

^a^SF = Shoot fly count (%); SB = Stem borer count (%); PHT = Plant height (cm); PNL = Panicle length (cm); PNW = Panicle width (cm); RNM = Rachis number; DTF = Days to 50% flowering (days); GRF = Grain filling duration (days); DTM = Days to 75% maturity (days); YLD = Grain Yield (kg/ha); HSW = 100 seed weight (g); Fe = Iron (ppm); Zn = Zinc (ppm); LSD = Least significant difference; SE± = Standard error of the mean diffference; Std = Standard deviation; CV = Coefficient of variation; ns = P > 0.05; ∗∗ = P ≤ 0.01; ∗∗∗ = P ≤ 0.001.

The six high grain Fe and Zn dense sorghum genotypes had a moderately high stem borer count (24.2–54.6%) and shoot fly count (2.8–13.4%) compared to the commercial check cultivar, SESO1 at 15.0% and 2.6%, respectively.

### 3.3 Genetic parameters for 13 quantitative traits in 345 sorghum genotypes evaluated in two locations in Uganda

The magnitude of GCV estimates was lower than the corresponding PCV estimates for the quantitative traits studied across locations (Table 7). High PCV value was recorded for shoot fly count (42.2%), grain yield (32.7%), panicle length (28.3%), 100 seed weight (27.7%), stem borer count (27.4%), plant height (26.4%), rachis number (26.3%), grain Zn (23.6%), and grain Fe (22.2%). Plant height, panicle length, panicle width, rachis number, days to 50% flowering, grain filling duration, and days to 75% maturity had relatively the same PCV and GCV values (Table 7). Some traits such as shoot fly count, stem borer count, and grain yield differed in their PCV and GCV estimates. High ECV estimates were recorded for shoot fly count and stem borer count.

**Table 7:** Genetic parameters for 13 quantitative traits in 345 sorghum genotypes assessed across two locations in Uganda.

Trait	Mean	PV	GV	EV	GCV	PCV	ECV	H^2^	GA	GA%
SF	4.4	0.7	0.3	0.5	25.7	42.2	33.5	37.0	0.7	32.2
SB	23.6	1.6	0.5	1.1	15.2	27.4	22.8	30.7	0.8	17.4
PHT	256.6	4577.7	4531.0	46.7	26.3	26.4	2.7	99.0	138.2	53.9
PNL	23.7	46.1	44.4	1.7	27.7	28.3	5.4	96.4	13.5	56.2
PNW	5.8	1.1	0.8	0.3	15.5	18.1	9.5	72.8	1.6	27.2
RNM	52	178.0	153.3	24.7	24.4	26.3	9.8	86.1	23.7	46.7
DTF	80.3	184.4	181.0	3.4	16.7	16.8	2.3	98.1	27.5	34.1
GRF	39.7	20.0	15.6	4.4	10.1	11.5	5.4	77.9	7.2	18.5
DTM	120.1	185.3	183.5	1.8	11.3	11.4	1.1	99.0	27.8	23.2
YLD	2909.3	925968.9	708783.5	217185.4	28.6	32.7	15.8	76.6	1519.6	51.6
HSW	2.1	0.4	0.3	0.1	24.4	27.7	13.1	77.6	1.0	44.3
Fe	37.5	69.6	37	32.6	16.2	22.2	15.2	53.1	9.1	24.4
Zn	18	18	11.5	6.5	18.8	23.6	14.2	63.7	5.6	31

^a^SF = Shoot fly count (%); SB = Stem borer count (%); PHT = Plant height (cm); PNL = Panicle length (cm); PNW = Panicle width (cm); RNM = Rachis number; DTF = Days to 50% flowering (days); GRF = Grain filling duration (days); DTM = Days to 75% maturity (days); YLD = Grain Yield (kg/ha); HSW = 100 seed weight (g); Fe = Iron (ppm); Zn = Zinc (ppm); PV = Phenotypic variance; GV = Genotypic variance; EV = Environmental variance; GCV = Genotypic coefficient of variation; PCV = Phenotypic coefficient of variation; ECV = Environment coefficient of variation; H^2^ = Broad-sense heritability; GA = Genetic advance; and GA% = Predicted genetic advance as percent of the mean.

Relatively low to moderate ECV estimates were computed for the assessed traits, ranging from 1.1% (days to 75% maturity) to 15.8% (grain yield) except in shoot fly count (33.5%) and stem borer count (22.8%). Most of the assessed quantitative traits had higher broad-sense heritability (63.7–99.0%) except for shoot fly count (37.0%), stem borer count (30.7%), and Fe content (53.1). High H^2^ (≥60%) was recorded for plant height, panicle length, panicle width, rachis number, days to 50% flowering, grain filling duration, days to 75% maturity, grain yield, 100 seed weight, and grain Zn content. Most assessed traits had higher GA% (23.2–56.2%) values except for grain filling duration, with moderate GA% (18.5%). Also, the stem borer count had moderate GA% at 17.4%. Fe content and shoot fly count with moderate H^2^ exhibited higher GA% at 24.4% and 32.2%, respectively).

### 3.4 Principal component and biplot analyses

**Figure 1:**
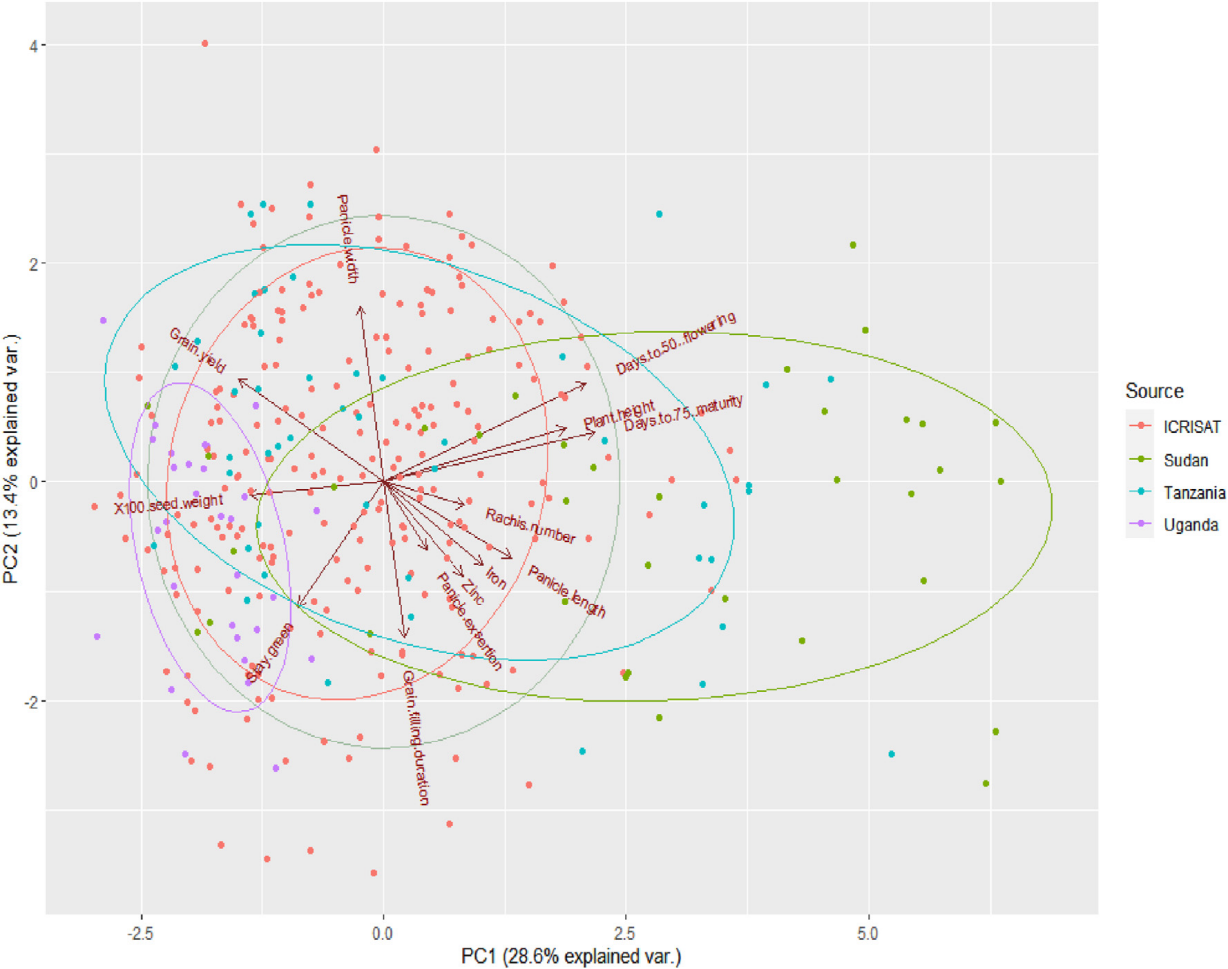
Principal component biplot showing the distribution of 334 sorghum genotypes and 13 quantitative traits assessed in two locations in Uganda.

The principal component analysis (PCA) results are summarised in Table 8. Six principal components (PCs) with eigenvalues greater than one accounted for 75% of the total variability. The first principal component (PC1) explained a variation of 29.0% followed by PC2 (13.0%) and PC3 (11.0%). The traits with a significant contribution in PC1 were days to 75% maturity, days to 50% flowering, plant height, grain yield, and 100 seed weight. While in PC2, most of the variation was contributed by panicle width, grain filling duration, and stay green. The highest positive contribution to the variation accounted for in PC3 was from panicle exsertion, panicle length, rachis number, and grain filling duration. High contribution to the variation in PC4 was attributed by grain Fe and Zn contents but in a negative dimension. The principal component biplots delineated the accessions into four groups according to the sources of germplasm collection (Figure 1). Accessions from Uganda were distinct from Sudan. The accessions from the furthest right side of PC1 were collections mainly from Sudan and Tanzania and grouped according to plant height, days to 50% flowering, days to 75% maturity days, Fe and Zn contents, panicle length, rachis number, and panicle exsertion.

**Table 8:** The six principal components (PC1 to PC6) and the respective eigenvalues for 13 contributing traits among 334 sorghum genotypes assessed in two locations in Uganda.

Parameters	PC1	PC2	PC3	PC4	PC5	PC6
Eigenvalues	1.93	1.32	1.17	1.03	0.99	0.96
Proportion of variance (%)	29	13	11	8	8	7
Cumulative variation (%)	29	42	53	61	68	75
	Eigenvalues (Loadings)					
Grain yield (kg/ha)	**-0.32**	0.29	0.26	-0.16	0.33	-0.09
100 Seed weight (gm)	**-0.30**	-0.04	0.27	0.13	**0.50**	0.08
Plant height (cm)	**0.40**	0.15	0.18	-0.01	0.12	0.27
Panicle length (cm)	0.28	-0.22	**0.42**	0.18	0.18	-0.30
Panicle width (cm)	-0.05	**0.50**	0.24	-0.26	0.19	0.34
Rachis number	0.18	-0.07	**-0.38**	0.12	**0.50**	-0.33
Days to 50% flowering (days)	**0.44**	0.28	0.02	0.11	0.07	-0.05
Grain filling duration (days)	0.05	**-0.44**	**0.37**	0.26	-0.02	0.33
Days to 75% maturity (days)	**0.46**	0.14	0.14	0.19	0.07	0.05
Grain Iron (ppm)	0.22	-0.24	0.18	**-0.60**	-0.22	0.13
Grain Zinc (ppm)	0.17	-0.27	-0.01	**-0.61**	0.33	-0.25
Panicle exsertion	0.10	-0.19	**-0.48**	-0.02	0.34	**0.63**
Stay green	-0.19	**-0.36**	0.18	0.04	0.16	0.08

^a^Boldfaced values denote important traits that contributed to the largest reliable variation in each principal component.

Ugandan accessions were grouped based on a higher 100 seed weight on the furthest left side of PC1 (Figure 1). ICRISAT accessions with high grain Fe, panicle length, rachis number, grain Zn, panicle exsertion, and grain filling duration were clustered in PC1. There was no definite delineation pattern between the accessions along the first two PC axes due to the overlap among the accessions in the biplot. The assessed quantitative traits were categorized according to the size of angles between dimension vectors (Figure 1). For example, plant height, days to 50% flowering, and days to 75% maturity had smaller angles between dimension vectors, thus high correlation among these variables. There was a moderate positive association between grain yield and 100 seed weight.

### 3.5 Cluster analysis

**Figure 2:**
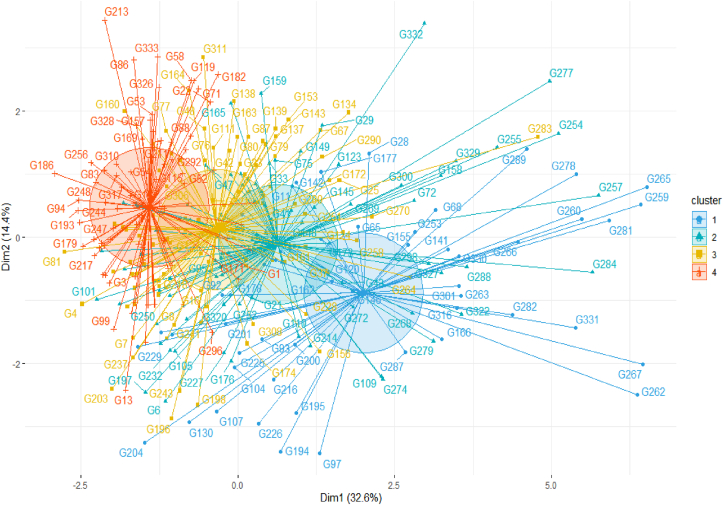
A scatter plot displaying 334 sorghum genotypes evaluated in two locations in Uganda based on 11 phenotypic traits. See Table 1 for the codes of genotypes and Table 9 for phenotypic traits.

Cluster analysis based on quantitative traits delineated the 334 genotypes into four genetic groups (Table 1, Table 9, and Figure 2). Most of the assessed genotypes (31.14%) were allocated in Cluster II, consisting of 71, 10, 12, and 11 germplasm from ICRISAT/Kenya, Sudan, Tanzania, and Uganda, respectively. This cluster comprised of genotypes with moderate grain yield (2397.4 kg ha^−1^), medium to late flowering (83.5 days), and tall plants (272.1 cm) with slightly higher grain Zn concentration (19.0 ppm) relative to other clusters. Cluster I with 90 accessions [(54 collections from ICRISAT/Kenya), Sudan (3), Tanzania (18), and Uganda (15)] consisted of genotypes with low grain yield (1484.5 kg ha^−1^), high rachis number (55.4), tall plants (286.6 cm), late maturity (129.8 days) and high Fe (42.4 ppm) and Zn (18.5 ppm) concentrations compared to genotypes in other clusters. Cluster I genotypes had a prolonged duration of grain filling (41.3 days) with late maturity (129.8 days).

**Table 9:** Summary of the cluster analysis showing the source of the assessed 334 sorghum germplasm collections, number of genotypes, and mean values for 11 phenotypic traits.

Variable	Cluster I	Cluster II	Cluster III	Cluster IV
	Number of genotypes			
Source of genotypes				
Kenya	54	71	35	63
Sudan	3	10	11	11
Tanzania	18	12	6	10
Uganda	15	11	1	3
Total	90	104	53	87
Traits				
Grain yield (kg/ha)	1484.5	2397.4	3247.0	4108.3
100 Seed weight (gm)	1.8	2.1	2.2	2.6
Plant height (cm)	286.6	272.1	245.2	229.4
Panicle length (cm)	27.1	24.2	23.3	22.5
Panicle width (cm)	5.2	5.7	5.9	6.4
Rachis number	55.4	52.4	48.6	47.6
Days to 50% flowering	88.5	83.5	78.8	74.4
Grain filling duration (days)	41.3	38.6	38.1	38.6
Days to 75% maturity (days)	129.8	122.1	116.9	113.0
Grain Iron (ppm)	42.4	36.8	38.0	34.8
Grain Zinc (ppm)	18.5	19.0	17.6	17.2

Cluster III had the least number of genotypes (53) characterized by moderate to high grain yield (with a mean of 3247.04 kg ha^−1^), medium plant height (245.2 cm) and short days-to-75% maturity (116.9 days), and moderate Fe concentration (38.0 ppm) and short grain-filling duration (38.1 days). Cluster IV had 87 genotypes with high grain yield (4108.3 kg ha ^1^), short plant height (229.4 cm), early maturity (113.0 days), low Fe (34.8 ppm) and Zn (17.2 ppm) concentration, low rachis number (47.6) with wide panicle width (6.4 cm) compared to genotypes in other clusters.

### 3.6 Correlation between yield and yield components and grain iron and zinc concentrations

The pair-wise correlation coefficients between the assessed traits of the 334 sorghum genotypes evaluated in two locations in Uganda is presented in Table 10. Grain yield had a moderate positive and significant association (P < 0.001) with 100 seed weight (r = 0.43) and panicle width (r = 0.37). Grain yield showed negative and significant association (P < 0.001) with days to 75% maturity (r = -0.43), days to 50% flowering (r = -0.36), and plant height (r = -0.31). There was a relatively low but significantly negative association between grain yield and Fe (r = -0.26) and Zn (r = -0.17) contents. A significant positive correlation was recorded between grain Fe and Zn contents (r = 0.32). There was a weak association between grain Fe/Zn concentration and other key sorghum yield components such as days-to-50% flowering, plant height, days-to-75% maturity, panicle length, panicle width, and 100 seed weight.

**Table 10:** Pearson correlation coefficients for 11 quantitative traits of 334 sorghum genotypes evaluated in two locations in Uganda.

Traits	PHT	PNL	PNW	RNM	DTF	GRF	DTM	Fe	Zn	HSW	YLD
PHT	-	**0.40∗∗∗**	0.12∗∗∗	0.08ns	**0.68∗∗∗**	0.05ns	**0.70∗∗∗**	0.26∗∗∗	0.18∗∗∗	-0.29∗∗∗	**-0.31∗∗∗**
PNL		-	-0.20∗∗∗	0.13∗	**0.36∗∗∗**	**0.30∗∗∗**	**0.46∗∗∗**	0.25∗∗∗	0.23∗∗∗	-0.10ns	-0.20∗∗∗
PNW			-	-0.08ns	0.07ns	-0.12∗	0.04ns	-0.04ns	-0.14∗	0.07ns	**0.37∗∗∗**
RNM				-	0.24∗∗∗	-0.03ns	0.24∗∗∗	0.01ns	0.16∗∗	-0.20∗∗∗	-0.22∗∗∗
DTF					-	-0.20∗∗∗	**0.95∗∗∗**	0.19∗∗∗	0.13∗	**-0.40∗∗∗**	**-0.36∗∗∗**
GRF						-	0.12∗	0.15∗∗	0.05ns	0.06ns	-0.20∗∗∗
DTM							-	0.23∗∗∗	0.14∗∗	**-0.38∗∗∗**	**-0.43∗∗∗**
Fe								-	**0.32∗∗∗**	**-0.27∗∗∗**	**-0.26∗∗∗**
Zn									-	-0.11ns	-0.17∗∗
HSW										-	**0.43∗∗∗**

^a^PHT = Plant height (cm); PNL = Panicle length (cm); PNW = Panicle width (cm); RNM = Rachis number; DTF = Days to 50% flowering (days); GRF = Grain filling duration (days); DTM = Days to 75% maturity (days); Fe = Iron content (ppm); Zn = Zinc content (ppm); HSW = 100 seed weight (g); YLD = Grain yield (kg/ha); ns = P > 0.05; ∗ = P ≤ 0.05; ∗∗ = P ≤ 0.01; ∗∗∗ = P ≤ 0.001.

## 4 Discussion

Sorghum is one of the principal cereal crops for food security in Africa and Asia and a source of raw material for the global beverage and syrup industry. Sorghum has multiple health benefits with products that are gluten-free and rich in phenolic compounds acting as antioxidants. Nutritionally enhanced sorghum is vital to reduce malnutrition among the rural and urban poor communities who depend on sorghum as their food staple. However, sorghum nutritional quality breeding has received little research and development support compared with other major staple crops such as maize and wheat. The genetic variation present in East African sorghum germplasm can be explored for quality traits and develop new varieties with farmer and market preferred product profiles. Therefore, this study was initiated to provide a comprehensive genetic diversity analysis to unravel the agronomic performance and nutritional quality traits among East African sorghum germplasm collections.

The present study found high diversity index of 0.92 for quality traits among the assessed 345 genotypes (Table 3). The magnitude of the diversity is higher than the mean diversity index reported by Desmae et al. (2016) at 0.67 in 974 landrace sorghum populations sampled from North-Eastern Ethiopia. The recorded genetic variability for quality traits was pronounced for grain color, stay green, panicle exsertion, and inflorescence type. Harlan and de Wet (1972) reported extensive variability in sorghum grain color, panicle exsertion, and inflorescence type. Farmers and breeders widely use these quality traits for selection in the region. Most germplasm collections (62.6%) assessed in this study had red and brown grain colors with loose inflorescence and drooping shapes. Sawadogo et al. (2014) reported that most (78.40%) of sorghum genotypes assessed in Burkina Faso had loose panicle shapes consistent with the current study. The predominantly red and brown grain color observed in >62% of the assessed sorghum genotypes indicates farmer preferences and long-term selection towards these traits. In a recent participatory rural appraisal study by Andiku et al. (2021), brown and light red grain sorghum cultivars were the most preferred traits by farmers. Sorghum flour prepared from brown or red grain blends well with cassava flour to prepare porridge locally referred to as *ugali*. White grain sorghum is mainly used for brewing. However, white grain types of sorghum are less preferred by farmers due to bird damage in the field and susceptibility to grain mold.

Most of the assessed genotypes had exerted inflorescence (50.5%) and well-exserted inflorescence (35.9%) (Table 3). Few sorghum genotypes (0.5%) had reduced leaf senescence and remained stay-green at maturity, indicating their potential for post-flowering drought tolerance breeding. Other qualitative traits that recorded wide genetic variability were glume cover (*H' =*0.92), and glume color (*H' =*0.72). A higher proportion (92.5%) of the tested genotypes were awnless at maturity, while 60% had their grains covered by red glume at 25%. This finding concurs with the studies of Sawadogo et al. (2014) in BurkinaFaso, Nadjiam (2021) in Chad, and Desmae et al. (2016) in Ethiopia, who reported that the majority of their germplasm collections had 25% glume cover. High glume cover is associated with poor threshing ability and reduced seed size (Desmae et al., 2016). The current study found glume color variation that can be selected and explored for grain mold resistance breeding. Sorghum genotypes with dark grain glumes are reportedly mold-resistant (Das et al., 2020). In the present study, a low level of genetic variation was recorded for leaf midrib (Table 3). White leaf midrib was present in 70.4% of the tested genotypes. This is contrary to Verma et al. (2017), who reported a high level of genetic variation for leaf midrib color among sorghum collections in India.

The test genotypes showed significant (P ≤ 0.05) variation for grain yield, nutritional quality traits, and quantitative agronomic traits (Table 4). This suggests that the germplasm pool harbor adequate genetic variation for breeding nutritionally enhanced and high-yielding varieties. Previous studies by Akatwijuka et al. (2016), Desmae et al. (2016), Kumar et al. (2012), Hariprasanna et al. (2014), Ng'uni et al. (2012) disclosed sufficient genetic variation for the quantitative traits in sorghum accessions sampled from Uganda, Ethiopia, India, and southern Africa. The high degree of genetic variation is attributed to the differences in their genetic constitution and the long selection history of the materials in various geographical locations in East Africa, including Kenya, Sudan, Tanzania, and Uganda.

Eighty genotypes expressed relatively higher grain yield (12.0–31.9%) compared with the best commercial check cultivar (NAROSORG3) (Table 5). These genotypes had early to medium maturity, short to medium plant height, and low grain Fe and Zn accumulation. Conversely, six genotypes (Tanzania Acc#42, Tanzania Acc#8, IS 3790, IS 30310, SUDAN COLL# 7, and IS 12750) were selected with relatively higher Fe and Zn concentrations (Table 6). The high levels of Fe and Zn contents in the present study agree with previous findings, including Kumar et al. (2013a), Hariprasanna et al. (2014), Ng'uni et al. (2012), and Reddy et al. (2010). Hence, these accessions are ideal candidates for Fe and Zn enhancement as donor parents. The grain yield of these genotypes was markedly low. Nevertheless, they can be used to introgress the nutritional quality genes into genotypes with high yield potential. The six Zn and Fe dense genotypes recorded moderately high stem borer and shoot fly damage indicating their susceptibility to insect pests (Table 6). Therefore, during nutritional quality breeding, multiple trait selection strategies should be pursued to enhance the genetic gains for yield and yield components.

The magnitude of PCV, GCV, heritability, and genetic advance is directly related to selection response. The extent of GCV estimates was lower than the corresponding PCV for all the quantitative traits (Table 7). The lower GCV than PCV estimates suggest a strong environmental influence on the expression of the phenotypic traits. Also, the PCV estimates for Fe and Zn concentrations were higher than their corresponding GCV. The high influence of the environmental variance suggests the need for genotype by environment interaction (GEI) analysis during nutritional quality trait improvement to select stable performing genotypes. Phuke et al. (2017) proposed that GEI assessment is key in selecting sorghum genotypes with stable Fe and Zn expression. The magnitudes of PCV and GCV values for plant height, panicle length, rachis number, days to 50% flowering, grain filling duration, and days to 75% maturity were relatively close, indicating the low environmental effects and allowing for direct selection for these traits (Semahegn et al., 2021). High H^2^ (≥60%) and GA% (23.2–56.2%) were recorded for grain yield and plant height, panicle length, panicle width, rachis number, days to 50% flowering, grain filling duration, days to 75% maturity, and 100 seed weight (Table 7). The high values for these traits offer a higher selection response. Gebregergs and Mekbib (2020) and Gebreyohannes et al. (2018) recorded high H^2^ estimates and GA% for grain yield, plant height, 100 seed weight, and panicle length among sorghum collections of Ethiopia. Also, high H^2^ (63.7%) and GA% (31.0%) were recorded for Zn concentration, indicating that this trait is probably under the influence of an additive gene effect, and presumably, its improvement could be achieved through targeted recurrent selection. Kumar et al. (2015) reported that Fe and Zn concentrations are highly heritable, under additive genetic control, and can be selected with a high grain yield. Other studies reported dominant gene action affecting Fe concentration in sorghum (Hariprasanna et al., 2014; Kumar et al., 2013b). Slightly low H^2^ (53.1%) and GA% (24.4%) were recorded for Fe concentration in the current study.

The PCA discerned important traits contributing to the largest reliable variability among genotypes. In this study, days to 75% maturity, days to 50% flowering, grain yield, 100 seed weight, plant height, panicle width, grain filling duration, and stay green were the most significant traits and accounted for the largest variation allocated in the first two PCs loadings (Table 8 and Figure 1). These traits have much influence on selection during crop improvement. Naoura et al. (2019), Mangena et al. (2018), and Abraha et al. (2015) reported that maturity days, days to 50% flowering, grain yield, 100 seed weight, plant height, panicle length, and stay green were highly influential traits and contributed the most to the total genetic variation in sorghum landraces. The PCA biplot classified the genotypes into four groups (Figure 1) based on the relationship between principal components, the phenotypic traits, and the genotypes. The assessed quantitative traits were categorized according to the size of angles between dimension vectors proposing a strong association among the traits (Mangena et al., 2018). For example, in the current study, a strong association was observed between plant height, days to 50% flowering, and days to 75% maturity; and grain yield and 100 seed weight. Accessions from Sudan and Tanzania were grouped on the right side of PC1 according to plant height, days to 50% flowering, days to 75% maturity days, grain Fe and Zn, panicle length, rachis number, and panicle exsertion (Figure 1). Conversely, Ugandan accessions were grouped based on a higher 100 seed weight on the left side of PC1. ICRISAT accessions were clustered in PC1 based on grain Fe and Zn, panicle length, rachis number, panicle exsertion, and grain filling duration.

The cluster analysis delineated the genotypes into four distinct categories (Table 9 and Figure 2), suggesting the presence of substantial genetic variation among the tested genotypes. Genotypes with similar agronomic performance were grouped irrespective of their sources of collection. For example, genotypes with low grain yield, high rachis number, tall plant height, late maturity, and high grain Fe concentration with moderate Zn concentration were grouped in Cluster I regardless of their origin (Table 9 and Figure 2). On the other hand, high yielding genotypes with short plant height, early maturity, and low Fe and Zn concentrations were allocated in Cluster IV. Genotypes with high Zn concentration and tall plant height with medium to late flowering days were assigned to Cluster II. This pattern of distribution could be related to seed exchange among farmers, research organizations, and non-governmental organizations in addition to cross-cutting agro-ecologies, cultures, and end uses in eastern Africa. Suitable parent selection with the trait of interest for crop improvement could be accomplished by integrating these records.

In the present study, a significant (P < 0.01) association was exhibited between grain yield and all the quantitative traits (Table 10). Grain yield had a moderate positive and significant association with 100 seed weight and panicle width. This implies that these traits can be improved concurrently through direct selection. However, there was a significant negative association between grain yield and Fe and Zn concentrations. Reddy et al. (2005) reported a significant negative association between micronutrients (i.e., Fe, and Zn) and grain yield in sorghum. A strong positive association was recorded between grain Fe and Zn contents. Previous studies: Hariprasanna et al. (2014); Kumar et al. (2012); Kumar et al. (2009); Kumar et al. (2010); Phuke et al. (2017); Reddy et al. (2010) reported a positive and significant association between grain Fe and Zn concentrations. The strong correlations between these two micronutrients suggest the possibility of concurrent improvement in these traits (Bhat et al., 2018; Kumar et al., 2013a). In the present study, days to 50% flowering, plant height, days to 75% maturity, and 100 seed weight had a weak association with grain Fe and Zn concentrations. This suggested that enhancing nutritional quality traits (i.e., Fe, and Zn) with farmer desired traits can be attained without compromising grain yield in sorghum. Interestingly, notable positive associations were recorded between agronomic traits that would allow simultaneous selection of these traits.

## 5 Conclusions

The current study found a high magnitude of genetic variations for grain yield and related traits, grain Fe and Zn concentrations among East African sorghum germplasm collections. Six genotypes (Tanzania Acc#42, Tanzania Acc#8, IS 3790, IS 30310, SUDAN COLL# 7and (IS 12750) with high grain Fe and Zn concentrations were identified as breeding parents for nutritional quality enhancement. Plant height, panicle length, panicle width, rachis number, days to 50% flowering, grain filling duration, days to 75% maturity, grain yield, 100 seed weight, and grain Zn concentration had relatively high heritability and genetic advance as percent of the mean. The present findings suggest that the identified traits are amenable to improvement through selection. The assessed sorghum germplasm was differentiated into four distinct genetic groups based on the cluster analysis. The study further found a significant positive association between grain Fe and grain Zn concentration (r = 0.32, P < 0.001) to pursue concurrent breeding for enhanced grain yield and the two nutritional traits in sorghum.

## Declarations

### Author contribution statement

Charles Andiku: Conceived and designed the experiments; Performed the experiments; Analyzed and interpreted the data; Wrote the paper.

Hussein Shimelis: Conceived and designed the experiments; Analyzed and interpreted the data; Wrote the paper.

Admire I.T. Shayanowako: Analyzed and interpreted the data; Wrote the paper.

Prakash I. Gangashetty; Eric Manyasa: Analyzed and interpreted the data; Contributed reagents, materials, analysis tools or data.

### Funding statement

This study was supported by 10.13039/100000865Bill and Melinda Gates Foundation [OPP1129015 and INV-009649_2020].

### Data availability statement

Data will be made available on request.

### Declaration of interests statement

The authors declare no conflict of interest.

### Additional information

No additional information is available for this paper.
